# Prevalence and Implementation of IAQ Programs in U.S. Schools

**DOI:** 10.1289/ehp.7881

**Published:** 2005-07-21

**Authors:** Dena Moglia, Alisa Smith, David L. MacIntosh, Jennifer L. Somers

**Affiliations:** 1 U.S. Environmental Protection Agency, Office of Air/Indoor Environments Division, Washington, DC, USA; 2 Environmental Health and Engineering Inc., Newton, Massachusetts, USA

**Keywords:** air, asthma, children’s health, environment, schools, survey

## Abstract

In this study, we determined the extent to which U.S. schools are implementing indoor air quality (IAQ) programs. We administered a questionnaire on IAQ programs and practices to a representative sample of schools. Participants were asked to provide information on the use, administration, implementation, challenges, and benefits of the IAQ program in their school. We developed an IAQ Practice Index to determine the level of activity directed toward IAQ in schools. The index was computed based on responses to specific survey questions and was normalized to a range of 0 to 100. Each question was weighted qualitatively according to its contribution to strong IAQ management practices. Forty-two percent of schools in the United States have an IAQ management program, and there has been sustained growth from 1998 through 2002 in the number of schools that have such programs. Nearly half of those schools use the U.S. Environmental Protection Agency’s IAQ Tools for Schools program. The IAQ Practice Index scores varied widely for schools with an IAQ management program, suggesting that having a program is not equivalent to implementing effective IAQ policies and procedures. Respondents indicated that their IAQ programs led to improved workplace satisfaction, fewer asthma attacks, fewer visits to the school nurse, and lower absenteeism. When actively supported by the school administration, an IAQ program appears to be a valuable factor in improving the learning environment for U.S. schoolchildren.

Schools are locations where children spend a large amount of their time, second only to time spent indoors at home. According to the U.S. Department of Education (DoE) National Center for Education Statistics (NCES) report *Condition of America’s Public School Facilities: 1999* (Lewis et al. 2000), about one-quarter of U.S. schools need extensive repair or replacement of one or more buildings. Nearly 11 million students attend these schools. Approximately 40% of schools report at least one unsatisfactory environmental condition such as poor ventilation, heating or lighting problems, or poor physical security (Lewis et al. 2000). Improper building operations and deferred maintenance contribute to poor indoor environmental conditions, affecting the levels of mold, mildew, dust, animal dander, radon, secondhand smoke, asbestos, and formaldehyde in schools (U.S. General Accounting Office 1995). These pollutants can affect indoor air quality (IAQ) and trigger various health symptoms, from headaches to allergies and asthma attacks (Samet and Spengler 1991).

The toll of these health conditions on education in America is large. Asthma alone accounts for 14 million missed school days each year (Centers for Disease Control and Prevention 2002). Asthma prevalence has been on a steep rise since 1980. Although many cases of asthma probably go undiagnosed, health officials estimate that 6.1 million children in the United States have asthma. Allergies are estimated to be the cause of an additional 2 million lost school days annually. Current evidence indicates that viral infections predispose children to asthma attacks and allergic responses (Papadopoulos and Johnston 2001). This is important, given that schoolchildren are estimated to experience 7–10 colds each year (Johnston and Holgate 1996) and that improved IAQ and ventilation may reduce the airborne transmission of viruses (Myatt et al. 2004). The effective management of IAQ in schools can reduce students’ exposure to the air pollutants that trigger allergies and asthma attacks, potentially improving students’ ability to learn.

There is strong research relating certain IAQ management practices with IAQ in buildings. For example, if you increase outdoor ventilation, all else being equal, indoor pollutant concentrations will decrease. This is the basis for national ventilation standards. Removing or managing sources of contaminants correspondingly decreases pollutant levels. The same can be said for air cleaning or filtration. Indeed, there are health and comfort relationships with such practices [see U.S. Environmental Protection Agency (EPA) 2004 for more information].

To gain information about the number of schools that have implemented IAQ management programs in our nation’s schools, the Office of Radiation and Indoor Air’s Indoor Environments Division of the U.S. EPA created the IAQ Practices in Schools Survey (U.S. EPA 2001). The IAQ Practices in Schools Survey examines the extent to which public and private schools nationwide have taken action to improve IAQ and implement an IAQ program.

## Materials and Methods

The IAQ Practices in Schools Survey included a representative sample in 2002 of all public and private schools that were operating in the United States during the 1999–2000 school year. The primary objective was to obtain a sample size sufficient to provide a reliable estimate of the fraction of schools throughout the United States that have implemented IAQ management practices, such as those recommended in the U.S. EPA’s Indoor Air Quality Tools for Schools (IAQ TfS) Action Kit program. A secondary objective was to obtain a sample with sufficient power to detect meaningful variation in IAQ management practices among schools.

Data used to identify the IAQ Practices in Schools Survey study population were obtained from the DoE’s NCES school registry, which features two separate databases, one for public schools and another for private schools. Both databases are updated on an annual basis. At the time that the questionnaire was developed, quality-assured public and private school data for the 1999–2000 school year were available from the Common Core of Data website (NCES 2002).

The eligibility criteria we established for public and private school data required that schools have a state postal code from one of the 50 states or the District of Columbia, were open during the reporting year of 1999–2000, and reported student enrollment > 0. Fundamental attributes of the public and private school data for 1999–2000 are summarized in Table 1. A total of 118,785 schools met the eligibility criteria for the combined data set. Public and private schools accounted for 75 and 25% of the eligible schools, respectively. Six percent of the public schools were omitted from the combined data set for failing to meet one or more of the eligibility criteria. All of the private schools met the eligibility criteria.

We employed a random sampling strategy, stratified by U.S. EPA region and school type, to distribute the IAQ Practices in Schools Survey (see Table 2 for a breakdown of states by U.S. EPA region). Sampling frequency was determined by the number of students in grades pre-kindergarten through 12. The percentage of schools sampled matched the percentage of schools in each of the 10 U.S. EPA regions stratified by public and private school system. Based on this strategy, 2,004 schools out of 118,785 eligible facilities were sampled. Sampling 2,004 schools, with an expected 50% return rate, yielded an anticipated sample size of 1,000 surveys. The sample of 1,000 schools was based on budget limitations. Precision and power analyses were conducted on the basis of 1,000 completed surveys. The sampling strategy had sufficient statistical power to detect a difference between different levels of a single variable, such as public and private schools, if one existed. However, this sampling strategy had insufficient power to detect a statistically significant difference between the 20 smaller strata of U.S. EPA region and school type. For this reason, conclusive analysis is limited to testing for differences on groups defined by different levels of a single variable, such as school type, rural versus urban school location, or school grade level. Differences found between the U.S. EPA regions and additional strata are discussed because they generate important hypotheses that will guide future research.

The IAQ Practices in Schools Survey was sent to 1,519 public and 485 private schools that met the eligibility criteria. Schools that did not return the survey within 3 weeks after distribution were called by telephone and prompted to complete and return the questionnaire. A total of 809 surveys were returned. The error rates for sample sizes of 1,000 and 809 are both within a 3% margin. Consequently, we determined that the sample size does not change the assumptions made in the sampling plan.

The four-page IAQ Practices in Schools Survey contains 11 multipart questions. Question 1 asks whether respondents use IAQ TfS, another IAQ management program, or none at all. Question 2 asks how long the plan has been in effect, using multiple-choice answers. Questions 3–7 ask respondents to rate multiple characteristics of their schools’ programs on a scale of 0 (none, not at all) to 5 (very much, excellent), as follows:

3. Please rate the quality and effectiveness of your school’s IAQ management plan (rate each item).

- A person is designated as IAQ coordinator/manager and has authority to carry out the IAQ management plan.
- The building and heating, ventilating, and air conditioning (HVAC) system receive regular maintenance to ensure that all systems are consistently functioning as designed.
- IAQ is a priority consideration for repairs and upgrades of the school building and HVAC system.
- The HVAC system consistently provides adequate control of temperature, humidity, and outdoor air ventilation to all occupied spaces.
- Art classes, industrial art classes, and science laboratories choose products and/or incorporate specific methods, e.g., exhaust ventilation, to minimize exposures of all students and staff to pollutants produced from these activities.
- Housekeeping (custodial) services maintain clean conditions in all areas.
- Cleaning products and methods are chosen to minimize exposure of students and staff to pollutants produced by housekeeping products.
- An IAQ walkthrough inspection and periodic checkups are used to monitor IAQ conditions and practices in the school(s).

4. Pest control in the school(s) is accomplished using the following (rate each item).

- Traps are used to monitor pest populations.
- Threshold targets are established for pest populations.
- Traps are used to kill and control pests.
- Hygienic conditions are strictly maintained to prevent infestations.
- Leaks, spills, condensation, and other moisture sources are strictly controlled.
- Pesticides are applied on a regular basis.

5. Your school administration supports the IAQ program.

6. School personnel actively participate in the IAQ program (rate participation by each group).

- Teachers
- Administrative staff
- Custodial staff
- Food service staff
- Health officers/school nurses
- Facilities and maintenance staff
- Other (specify)

7. Please rate the extent to which the following have been barriers to implementing IAQ practices in your school(s).

- Potential liability
- Costs
- Lack of resources
- Lack of knowledge
- Competing priorities
- School administration
- School board Question 8 asks for the outdoor air ventilation rate in respondents’ schools, using multiple-choice options.

Question 9 asks which of the various IAQ TfS checklists have been distributed, specifying that respondents should check all that apply. Question 10 asks what percentage of those checklists have been completed and returned, using multiple-choice options. (This question helps gauge the extent to which the entire school team participates in IAQ management.) Question 11 asks respondents to offer their opinions as to whether their IAQ program has led to lower absenteeism, better test scores, increased productivity, fewer asthma episodes, improved workplace satisfaction, or fewer visits to the health officer or school nurse. Respondents are instructed to check all that apply for question 11.

We designed an IAQ Practice Index as a way to quantify the extent of each school’s IAQ management practices. It is a tool to facilitate the use and interpretation of the survey results. An IAQ Practice Index score for each respondent was computed from responses to questions 3, 4, 5, 6, and 8. Questions 1 and 2 were excluded from the index because they are not quantitative questions; however, the way in which these questions relate to the IAQ Practice Index is addressed in the analysis. Questions 7 and 11 were excluded because they do not provide an absolute measure of implementation. Rather, they offer insight into factors that the investigators expected to correlate with the strength of IAQ management practices. Questions 9 and 10 were excluded because they relate specifically to IAQ TfS, whereas the index is intended to measure management practices regardless of the program employed.

We assigned values to index questions based on our professional assessment of the qualitative importance of each question’s contribution to good IAQ management practices. Table 3 depicts the IAQ Practice Index scoring methodology.

We determined that responses to the five IAQ Practice Index questions must be at least 80% complete for a survey to be included in the index. This completeness criterion was established based on the fact that non-responses were given scores of zero when computing the index. Thus, incomplete surveys would almost necessarily receive a lower IAQ index score than would complete surveys. We chose this approach, rather than computing the index from only those questions on any given survey that received complete responses, because we felt that basing the index on only a subset of IAQ metrics would yield invalid results. There is a fairly strong correlation (Spearman *r* = 0.45) between completeness and index scores across all 809 schools. However, the correlation becomes quite weak (Spearman *r* = 0.18) for only schools with completeness > 80%. Thus, applying the 80% completeness criterion affords characterization of IAQ management practices across schools with minimal potential bias from incomplete questionnaires.

In addition, completeness dropped off precipitously < 80%. This means that the study would have had to include substantially incomplete questionnaires to gain even a modest increase in sample size for the IAQ index. For example, to add even an additional 100 schools to the sample size (a 17% increase over the 587 schools), the study would have had to include completeness percentages as low as 55%. We determined that the limited amount of information provided from schools with completeness < 80% could not be considered a valid indicator of their IAQ management practices.

## Results

### Quality assurance.

A total of 809 completed questionnaires were returned for an overall survey response rate of 40%. There was no evidence of systematic error or selection bias associated with the response rate. The distribution of returned and targeted questionnaires was similar with respect to the stratification criteria of geographic region and public/private schools. Academic resource, demographic, and socioeconomic characteristics of schools that returned the questionnaire were approximately equal to those of schools that did not return it. IAQ management practices were independent of the amount of follow-up effort required to elicit return of a questionnaire.

Seventy-two percent (586 of 809) of the returned questionnaires met our completeness criterion of 80% for inclusion in the IAQ Practice Index calculation. A total of 2,004 surveys were mailed to schools. Thus, the questionnaire completion rate used to calculate the index was 29% (586 of 2,004 questionnaires).

### Prevalence of IAQ programs.

Forty-two percent of the 809 schools that responded to the questionnaire had an IAQ management program, and 20% used U.S. EPA’s IAQ TfS program. Thirty-six percent of schools with an IAQ management program had an IAQ plan in place for > 5 years, 22.5% of schools for 2–4 years, 19.6% of schools for 1–2 years, and 13% of schools for < 1 year.

IAQ programs do not appear to be distributed evenly between public and private schools. The survey results indicate that nearly 50% of public schools across the nation have a program to manage IAQ. However, only 20% of private schools appear to have an IAQ program.

**Figure 1:**
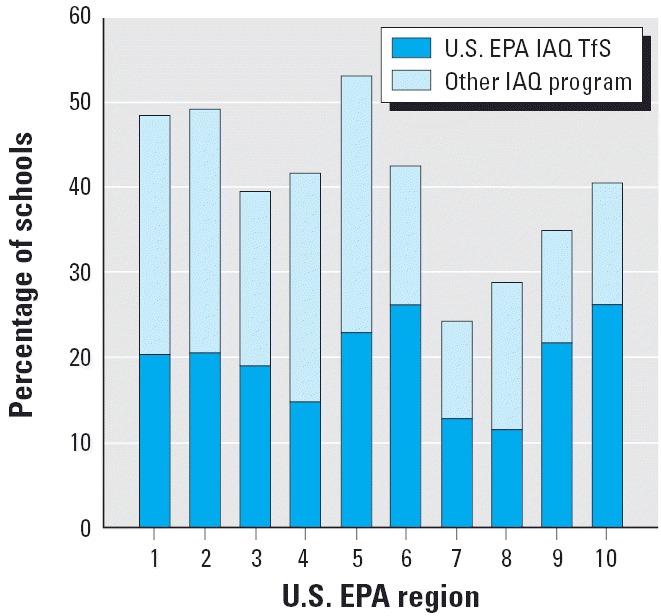
Percentages of schools using the U.S. EPA’s IAQ TfS program or another IAQ management program across the U.S. EPA’s 10 geographic regions in the United States.

The percentage of schools in each U.S. EPA region with an IAQ management program is presented in Figure 1. The portion of schools using U.S. EPA’s IAQ TfS is distinguished from schools that use a different IAQ management program. The plot shows that at least 40% of the schools in U.S. EPA regions of the eastern United States have an IAQ management program, whereas < 40% of the schools in the U.S. EPA regions of the western United States have an IAQ management program.

With regard to IAQ TfS, distribution and use of program checklists are important indicators of IAQ program implementation. The administration, ventilation, building maintenance, and walkthrough checklists were distributed to staff in more than half of the schools that use IAQ TfS. The waste management checklist was the least frequently distributed checklist. Approximately one-fifth of schools that use IAQ TfS reported that 57.3% of the IAQ checklists had been completed and returned. In comparison, the remaining four-fifths of schools that reported not using a management plan or using a plan other than IAQ TfS reported that 7.8% and 12.8% of the IAQ checklists had been completed and returned, respectively.

Nearly three-quarters (73.2%) of the schools with an IAQ management program report receiving substantial support for the program from their school administration (based on a rating of 4 or 5 for question 5). Among schools with active IAQ management programs, facilities, maintenance, and custodial staff were active participants in nearly 80% of the programs (based on responses to question 6). Food service, health care, and administrative staff were also active participants in the school’s IAQ programs. This indicates a strong measure of engagement among all members of a school community, which is an important aspect of a well-functioning IAQ management program.

### IAQ Practice Index.

**Figure 2:**
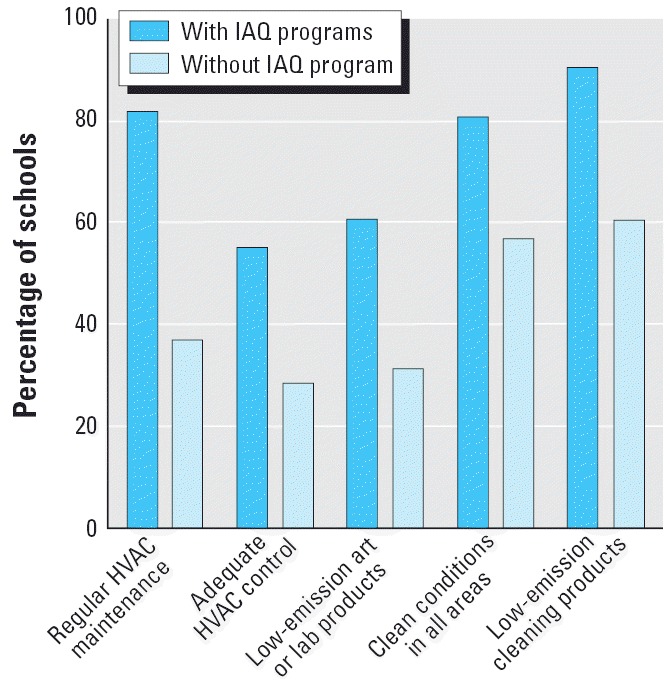
Percentages of schools using an IAQ program and not using an IAQ program: five parameters affecting IAQ policies and procedures.

The IAQ Practice Index ranges from a minimum possible score of 0 to a maximum of 100. The survey results revealed that the quality and effectiveness of IAQ management programs varied widely, from 20.6 to 100, as measured by the index. Given our expertise in IAQ management, we determined that a score of 70 would be used as a baseline, indicating a well-functioning IAQ program consistent with U.S. EPA guidance. Of the schools with an IAQ management program, 57% had a score > 70. A comparison of schools that have an IAQ program to those without an IAQ program on five parameters affecting IAQ policies and procedures is presented in Figure 2.

**Figure 3:**
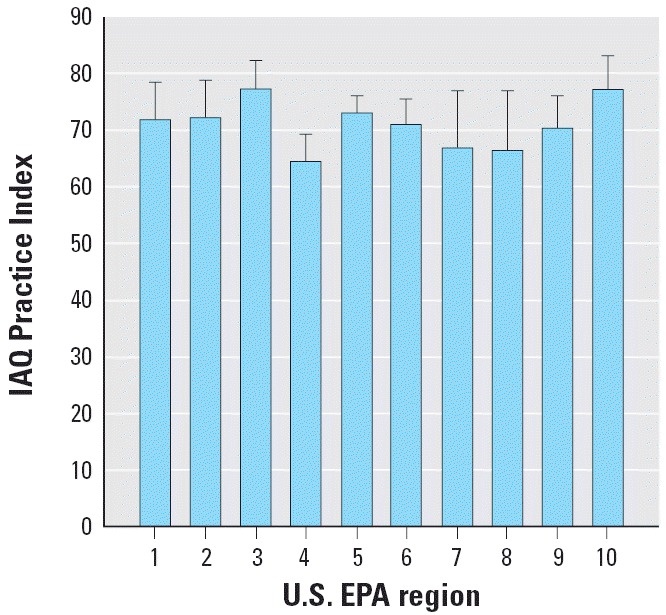
IAQ Practice Index (mean ± SE) across the U.S. EPA’s 10 geographic regions for those schools reporting use of an IAQ management program and completing at least 80% of the survey.

The mean IAQ Practice Index across U.S. EPA regions ranged from 64.4 in Region 4 (Southeast) to 77.1 in Region 10 (Upper Northwest). Mean IAQ Practice Indices varied significantly (*p* = 0.0307) among U.S. EPA regions according to the results of a one-way generalized linear model, although the differences across regions are < 15 index units (see Figure 3 for further regional comparisons). More observations from schools in Regions 7 and 8 are needed to explore spatial variability of IAQ management programs in schools more fully (see Table 2 for further regional statistics).

The mean IAQ Practice Index did not differ significantly (*p* = 0.7746) between the 287 public schools (mean = 70.8) and 31 private schools (mean = 71.7) that met the completeness criterion for scoring and calculation of the index.

Questionnaire respondents were asked their opinion on whether their IAQ program led to any associated benefits. Improved work-place satisfaction was the most frequently reported benefit of an IAQ program among schools that have an IAQ program. Improved health status of students, as indicated by fewer asthma episodes, fewer visits to the school nurse, and lower absenteeism, was reported by 28–33% of schools that have an IAQ program. Cost, lack of resources or knowledge, and competing priorities were the most frequently reported barriers to implementation of an IAQ program among the schools that do not have a program.

## Discussion

The IAQ Practices in Schools Survey was the first national assessment of IAQ management programs in U.S. schools. The survey yielded unique information about management of factors that influence IAQ in schools and provided a basis for evaluation of the status and trends of school IAQ management programs.

The principal limitations of the survey are associated with the mechanism chosen to administer the questionnaire, certain details of the questionnaire format and wording, and the potential for self-selection bias.

The survey consisted of a self-administered questionnaire that was addressed to the “school official.” School representatives with 350 different job titles completed and returned the survey, although principals represented the bulk of respondents at 33.6%. The next most frequently reported job title represented only 6.4% of the respondents. School officials with different job titles and responsibilities may have different amounts of information about IAQ management programs in schools and also may hold different perspectives about the importance and role of IAQ management programs in schools. We analyzed the survey responses and found no evidence that principals responded differently to the questionnaire than other categories of respondents. This suggests that if any bias was present based on the respondent’s position within the school, it is unlikely to have had an impact on the analysis.

Another result of the self-administered feature of the survey is that respondents had limited ability to resolve questions about the intent and meaning of instructions, queries, and answers included in the questionnaire. The distribution of responses to certain questions is evidence of apparent confusion on the part of some respondents. For example, 404 schools reported that they do not use an IAQ management program (question 1), yet 20% of those same schools reported that their IAQ management plan had been in use for < 1 year to > 5 years. The distinction between an IAQ management program and IAQ management plan may not have been clear to all of the respondents.

The internally inconsistent responses are in part likely the result of potentially ambiguous instructions and questions in selected portions of the questionnaire. For example, questions 3, 5, 6, 7, and 11 are queries about various aspects of a school’s IAQ management program. One might anticipate that only schools with an IAQ management program would respond to those questions. Indeed, some respondents who checked “None” for question 1 (i.e., they do not have an IAQ program) did not complete the remainder of the questionnaire. However, many schools without an IAQ management program did respond to these questions with an answer other than “None.” Responses to these questions by schools without an IAQ management program are difficult to interpret. In future surveys, the instructions on the questionnaire will stipulate whether responses to certain questions are conditional on responses to preceding questions.

The wording of select questions also may have been a source of confusion for schools rating their IAQ management practices. For example, question 7 asks the school to rate the extent to which potential liability, costs, lack of resources, and other factors have been barriers to implementing IAQ practices. There are two possible interpretations of the rating scale: A factor could be construed as a “poor” or otherwise weak attribute (rating of 0 or 1) of the school’s IAQ program, or its significance as a barrier could be construed as “a lot” or “very much” (rating of 4 or 5). The bimodal distribution of the relationship between the IAQ Practice Index and responses to question 7 supports the idea that interpretation of the rating scale for this question differed among schools.

Finally, the IAQ Practices in Schools Survey identified a wealth of data on IAQ management in schools. However, the relationship between implementation of IAQ management practices and actual IAQ in schools cannot be addressed by questionnaire. The prospect of obtaining quantitative measures of IAQ in conjunction with detailed information on IAQ management practices is a consideration for future programmatic efforts.

The survey results indicate that many public schools in the United States have adopted IAQ management programs. Fifty percent of U.S. public schools have some sort of IAQ program. With only a 20% adoption rate, private schools, on the other hand, have room for improvement with respect to their IAQ management practices. The reasons for this disparity will be an interesting topic of future research. The northwestern United States has schools most involved in their IAQ management programs, scoring the highest average IAQ Practice Index in the country, with the mid-Atlantic region scoring a close second. The Great Lakes region has the highest percentage of schools with an IAQ program. Areas of the country where IAQ has received less attention include the Southeast, which had the lowest IAQ Practice Index, and the midwestern states, which have both a low IAQ Practice Index score and the fewest schools with an IAQ management program.

The breadth of questions covered in the questionnaire provides a way to quantify the quality of overall management practices for schools that have an IAQ management program. The central tendency of the IAQ Practice Index indicates the typical level of activity directed toward IAQ in schools, whereas the dispersion of the IAQ Practice Index describes the variability in activity of IAQ programs across schools. The quality and effectiveness of IAQ management programs, as measured by the IAQ Practice Index, varied substantially among schools. Assuming that the index accurately reflects the extent of IAQ program implementation, this finding implies that the use of an IAQ management program is not equivalent to implementing effective policies and procedures that proactively and effectively manage IAQ issues. We believe that additional outreach efforts may effectively improve the IAQ of schools in the United States, but further research would be required to support this assumption.

The sample size provided sufficient statistical power to identify relationships between IAQ management practices (measured by the IAQ Practice Index) and factors such as administrative support and authority to implement the program. Notably, the IAQ Practice Index was positively correlated with the reported level of administration support for a school’s IAQ management program and the designation of a manager or coordinator to implement the program. Thus, if a school’s administration was reported to support the school IAQ management plan and there is a designated IAQ manager/coordinator, the school was more likely to have an effective, higher-quality IAQ management program. The relationships between the IAQ Practice Index and selected questions were evaluated for questionnaires that were at least 80% complete. The analyses were repeated using completeness criteria of 90% and 100%, and there were no appreciable changes in the results. Thus, the findings are robust with respect to the choice of 80% completeness criterion.

A total of 809 completed questionnaires were returned, for a survey response rate of 40%. The survey was designed with a 50% response rate. Because of the lower than anticipated response rate, we conducted two sets of analyses to address the potential for substantive bias in the survey results.

In the first set of analyses, we examined the completed questionnaires for indications that the follow-up effort required for a school to return the questionnaire is associated with IAQ management practices. The assumption is that less follow-up effort indicates greater interest in IAQ and that interest in IAQ is associated with IAQ management practices. If response time is not associated with IAQ management practices, then one reason for concern about the potential for self-selection to bias the survey results would be eliminated. We measured follow-up effort by the number of telephone calls made to the school before the questionnaire was returned. The percentage of schools with an IAQ management program and the IAQ Practice Index varied little among levels of follow-up effort. In addition, the number of follow-up telephone calls was not correlated with the percentage of schools with an IAQ management program (Spearman *r* = 0.33, *p* = 0.2238), completeness (Spearman *r* = 0.37, *p* = 0.4685), or IAQ index (Spearman *r* = 0.09, *p* = 0.8717). These results suggest that IAQ practices in schools that did not return the questionnaire and presumably required more intensive follow-up are not substantively different from schools that did return the questionnaire. These data suggest that the survey results are not influenced by self-selection bias.

The second set of analyses consisted of two phases. First, characteristics of schools that returned the questionnaire were compared with characteristics of schools that did not return the questionnaire. The assumption is that certain school characteristics that were not included in the survey design such as stratification variables may be associated with IAQ management practices. In that case, differences in such characteristics between responder and nonresponder schools could indicate bias in the survey results. Second, for characteristics that differed substantially between responder and nonresponder schools, we assessed the relationship between those characteristics and IAQ management practices to evaluate the magnitude of potential bias.

Academic resource, demographic, and socioeconomic characteristics of schools that returned the questionnaire were approximately equivalent to those of schools that did not return the questionnaire, as shown in Table 4. In addition, IAQ management practices as measured by the IAQ Practice Index did not vary with respect to socioeconomic or demographic attributes of schools or their student populations, including Title I (federal program for economically disadvantaged children who reside in areas with a high concentration of low-income families) status, grade level, enrollment, rural/nonrural location, median household income, or percentage of students eligible for free or reduced-price lunch programs. These results indicate that schools in less affluent areas are as likely to have an IAQ management program as are schools in other areas and suggest that school size and financial resources are not important determinants of a school’s ability or willingness to implement an IAQ management program. In part, this may reflect the fact that many IAQ practices are low-cost activities.

Of the 124,288 public and private schools in the NCES databases, 30,645 (24.7%) are in rural locations. Thus, the survey results over-represent rural schools by approximately 17%. This overrepresentation is of consequence only if IAQ management practices differ between rural and nonrural schools. Mean completeness and IAQ index agreed well for rural (82% and 51.3, respectively) and nonrural schools (81% and 54.7) that returned a questionnaire. Based on these findings, there does not appear to be a significant difference in IAQ management practices between rural and urban schools. The percentage of rural schools with an IAQ program was 37% (87 of 238), whereas 45% (255 of 571) of nonrural schools had a program. Overall, 42.3% [95% confidence interval (CI), 38.9–45.7%] of schools that returned a questionnaire had an IAQ program. We devised an adjusted estimate by weighting the proportion of the rural and nonrural schools with an IAQ program by the national distribution of rural and nonrural schools. After this adjustment, 42.7% of schools nationwide are estimated to have an IAQ program. The adjusted estimate is nearly equal to the original estimate and is within the 95% CI derived from the raw survey data.

A particularly encouraging result is that 50% of the public schools surveyed reported use of an IAQ management program. The information on the number of years that an IAQ management program has been in place suggests that there has been a sustained increase in the use of IAQ management programs over time. The survey results indicate that schools are paying an increasing amount of attention to IAQ.

## Conclusion

An estimated 42.3% of schools in the United States have an IAQ program, and the use of IAQ management programs in schools has increased from 1998 through 2002. Variation in the IAQ Practice Index indicates that having an IAQ management plan is not equivalent to implementation of effective IAQ policies and procedures. When actively supported by the school administration, an IAQ program is reported to be a valuable factor in improving the learning environment for U.S. schoolchildren.

## Figures and Tables

**Table 1:** Attributes of the NCES public and private school data sets [no. (%)] for the 1999–2000 school year.

Attribute	Public schools	Private schools
Schools in database	95,289 (100)	28,939 (100)
School not in 50 states or D.C.	1,849 (2)	0 (0)
Closed school	2,034 (2)	0 (0)
School did not report enrollment	533 (1)	0 (0)
School enrollment = 0	1,059 (1)	0 (0)
Schools not meeting eligibility criteria	5,443 (6)	0 (0)
Schools meeting eligibility criteria	89,846 (94)	28,939 (100)

**Table 2:** IAQ Practice Indices across the 10 U.S. EPA regions for schools with an IAQ management program.^a^

Region	States	Frequency	IAQ Practice Index^b^	Schools with index ≥ 70 (%)
1	Connecticut, Maine, Massachusetts, New Hampshire, Rhode Island, Vermont	25	71.8	64
2	New Jersey, New York, Puerto Rico,^c^ U.S. Virgin Islands^c^	28	71.9	57
3	Delaware, Maryland, Pennsylvania, Virginia, West Virginia, District of Columbia	21	77.0	71
4	Alabama, Florida, Georgia, Kentucky, Mississippi, North Carolina, South Carolina, Tennessee	46	64.4	41
5	Illinois, Indiana, Michigan, Minnesota, Ohio, Wisconsin	87	73.0	60
6	Arkansas, Louisiana, New Mexico, Oklahoma, Texas	40	70.7	60
7	Iowa, Kansas, Missouri, Nebraska	15	66.7	53
8	Colorado, Montana, North Dakota, South Dakota, Utah, Wyoming	13	66.3	54
9	Arizona, California, Hawaii, Nevada, Pacific Islands,^c^ Tribal Nations subject to U.S. law	27	70.1	56
10	Alaska, Idaho, Oregon, Washington	16	77.1	81

a The total number of schools that report having an IAQ management plan with a questionnaire completion rate of > 80% is 318.

b Mean IAQ Practice Indices varied significantly (*p* = 0.0307) among U.S. EPA regions.

c Schools that were not in the 50 states or the District of Columbia were excluded from this study.

**Table 3:** IAQ Practice Index scoring methodology.

Question	Value	Scoring/ranking methodology
Question 3	30	Determine the average score for subquestions *a*–*h* and divide by 5 (the range of possible answers). Multiply this total by the assigned weight, 30.
Question 4	10	Create a filter for this question. If a respondent answers subquestion *f* with a response of 0 or 1, then total the average score of subquestions *a*–*e* and divide by 5. Multiply the total by the assigned weight, 10. However, if a respondent answers subquestion *f* with a response of 2–5, then divide the total average score of subquestions *a*–*e* in half; divide by 5; and multiply by the assigned weight, 10.
Question 5	25	Divide the score by 5 and multiply the total by the assigned weight, 25.
Question 6	25	Determine the average score for subquestions *a*–*g*, divide by 5, and multiply by the assigned weight, 25.
Question 8	10	The following values have been assigned to each subquestion:*a*) No particular setting = 0*b*) < 5 cfm per occupant = 0*c*) 5–10 cfm per occupant = 3*d*) 11–14 cfm per occupant = 7*e*) ≥ 15 cfm per occupant = 10

cfm, cubic feet per minute.

**Table 4:** Selected characteristics of schools that did (*n* = 809) and did not (*n* = 1,195) return the IAQ Practices in Schools Survey.

	Returned questionnaire			Did not return questionnaire		
		Interquartile range			Interquartile range	
Characteristic	Median	Q1^a^	Q3^b^	Median	Q1^a^	Q3^b^
Student:teacher ratio	15.4	12.6	18.1	15.5	12.9	18.8
Enrollment (no. of students)	392	200	645	376	144	637
Median household income ($)^c^	38,676	32,171	50,729	39,156	31,292	51,336
Free lunch program (%)^d^	23	10	41	27	12	52
Reduced-price lunch program (%)^e^	7	4	11	7	4	11

a First quartile (Q1) is the 25th percentile of distribution.

b Third quartile (Q3) is the 75th percentile of distribution.

c Median household income for ZIP code of school.

d Percentage of school students eligible for the free lunch program.

e Percentage of school students eligible for the reduced-price lunch program.
